# Outcomes of Extracorporeal Life Support (ECLS) in Acute Severe Asthma: A Narrative Review

**DOI:** 10.1007/s00408-023-00667-x

**Published:** 2024-03-21

**Authors:** Nneoma Ekechukwu, Sachin Batra, Deborah Orsi, Marjan Rahmanian, Maneesha Bangar, Amira Mohamed

**Affiliations:** Montefiore Medical Center and Albert Einstein College of Medicine, Bronx, NY USA

**Keywords:** Status asthmaticus, Acute severe asthma, Extracorporeal membrane oxygenation (ECMO), Extracorporeal carbon dioxide removal (ECCO2R), Extracorporeal life support

## Abstract

**Background:**

In this narrative review we aimed to explore outcomes of extracorporeal life support (extracorporeal membrane oxygenation (ECMO) and extracorporeal carbon dioxide removal (ECCO2R)) as rescue therapy in patients with status asthmaticus requiring mechanical ventilation.

**Methods:**

Multiple databases were searched for studies fulfilling inclusion criteria. Articles reporting mortality and complications of ECMO and ECCO2R in mechanically ventilated patients with acute severe asthma (ASA) were included. Pooled estimates of mortality and complications were obtained by fitting Poisson’s normal modeling.

**Results:**

Six retrospective studies fulfilled inclusion criteria thus yielding a pooled mortality rate of 17% (13–20%), pooled risk of bleeding of 22% (7–37%), mechanical complications in 26% (21–31%), infection in 8% (0–21%) and pneumothorax rate 4% (2–6%).

**Conclusion:**

Our review identified a variation between institutions in the initiation of ECMO and ECCO2R in patients with status asthmaticus and discrepancy in the severity of illness at the time of cannulation. Despite that, mortality in these studies was relatively low with some studies reporting no mortality which could be attributed to selection bias. While ECMO and ECCO2R use in severe asthma patients is associated with complication risks, further studies exploring the use of ECMO and ECCO2R with mechanical ventilation are required to identify patients with favorable risk benefit ratio.

## Introduction

Status asthmaticus/acute severe asthma (ASA) is characterized by severe expiratory air flow limitation leading to hypercapnic and hypoxic respiratory failure and carries a mortality rate as high as 7% despite mechanical ventilation [1]. Extracorporeal membrane oxygenation (ECMO) and extracorporeal carbon dioxide removal (ECCO2R) can act as rescue therapies in the subset of patients in whom severe hyperinflation persists, potentially causing barotrauma and hemodynamic instability.

The underlying rationale for the use of these extracorporeal therapies is usually as a bridge to either lung recovery by optimizing lung mechanics and allowing ultra-lung protective ventilation without impairing gas exchange or as a bridge to lung transplant if recovery is unlikely. Common indications for these therapies are acute respiratory distress syndrome (ARDS) or interstitial lung disease (ILD) [2], however, evidence to support its role in ASA is limited to a few case studies and retrospective registries [3].

**Fig. 1:**
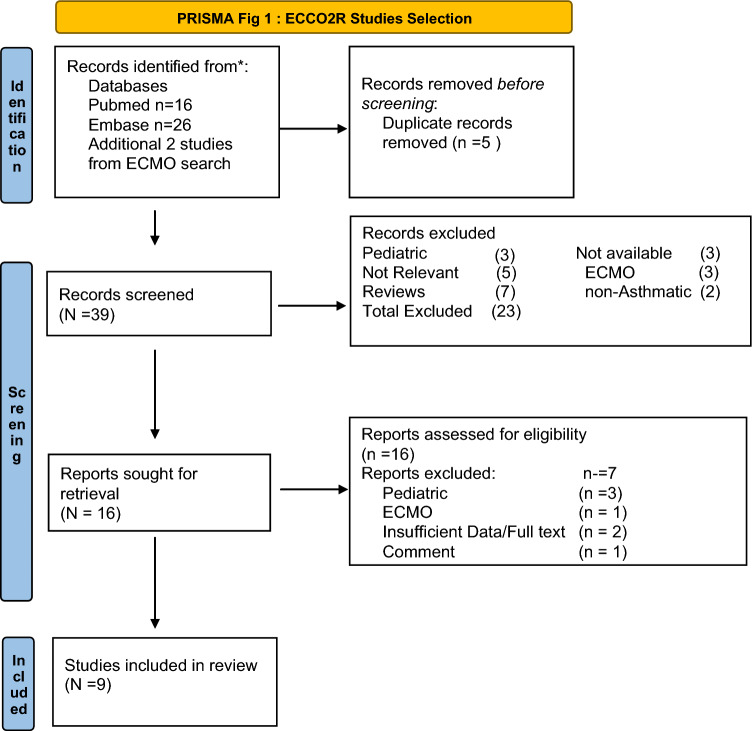
ECCO2R studies selection

**Fig. 2:**
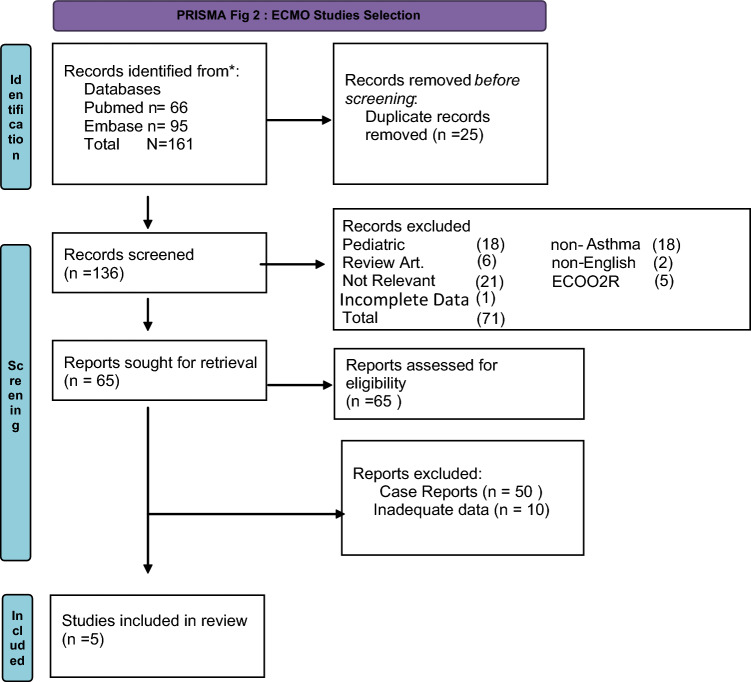
ECMO studies selection

We reviewed the literature to generate pooled estimates of mortality and complications of ECMO and ECCO2R therapy in patients with ASA on mechanical ventilation (Figs. 1 and 2).

## Methods

### Design

We systematically searched for publications reporting the use of ECMO and/or ECCO2R in ASA. We searched electronic databases Pubmed and EMBASE for studies published before March 2023. PubMed was queried using MeSH (Status Asthmaticus) and ECMO. Embase was queried using the EmTree term {(“Asthma” OR its synonyms)} AND {(“Extracorporeal membrane oxygenation” OR its synonyms)}. Similarly, for ECCO2R studies MESH terms (Status Asthmaticus) and ECCO2R. Embase was searched using EmTree term {(“Asthma” OR its synonyms)} AND {(“ECCO2R” OR its synonyms)}. Search results were exported and combined. After duplicate articles were removed, titles and abstracts of the remaining were reviewed and selected for review if deemed relevant to the study. Full-text manuscripts of these articles were reviewed, and selection was based on inclusion and exclusion criteria.

Inclusion criteria were reports of adult patients with status asthmaticus managed with ECMO and/or ECCO2R, while exclusion criteria included review articles, pediatric studies, and articles that were published in a language other than English. Additionally, for ECMO studies inclusion was limited to case series or registry data. Two independent authors assessed eligibility criteria and abstract selection. A full-text review was done for studies that met inclusion criteria.

We performed a review of the literature to estimate pooled mortality and complications using Poisson’s normal modeling.

## Results

Five ECMO studies and eight ECCO2R studies reporting a total of 494 patients were identified (Table 1). All the ECMO studies were retrospective reviews [1, 4–7] and the ECCO2R studies were mostly case reports, with only one being a case series [3, 8–15]. After exclusions, five ECMO studies and one ECCO2R study were included in total. Most studies included utilized VV circuits. One study used both VV and VA ECMO in status asthmaticus [5]. The six studies included showed pooled mortality estimates 13.7% (95% CI: 9.5–19.8%).

**Table 1:** Study population: pre-ECMO

Study	Intervention	*N*	PEEP	Total PEEP	PIP	P-Plat	DP	pH	pCO_2_	FiO_2_	PF ratio
Yeo, 2017 [1]	ECMO	272	8 ± 6	NA	38 ± 11	NA	29 ± 30	7.1 ± 0.2	81 ± 51	81.2 ± 23.0	NA
Zakrajsek, 2023 [5]	ECMO	127	NA	NA	NA	NA	NA	NA	NA	NA	NA
Patel, 2020 [6]	ECMO	22	NA	NA	NA	NA	NA	7.12 ± 0.2	96 ± 31	NA	NA
Mikkelsen, 2009 [7]	ECMO	24	7 ± 3	NA	39 ± 9	NA	NA	7.17 ± 0.16	120 ± 58	NA	244 ± 180
Di Lasco, 2017 [4]	ECMO	16	4 ± 0	NA	53 ± 12	NA	NA	6.89 ± 0.0	111 ± 4	NA	71 ± 123
Bromberger, 2020 [3]	ECCOR	26	13 (9–14)^a^	NA	53 (45–63)	20 (16–25)	NA	7.13 (6.97–7.20)	93 (71–128)	50 (40–70)	248 (181–350)

*PIP* peak inspiratory pressures, *PEEP* positive end expiratory pressure, *P-Plat* plateau pressures, *DP* driving pressures

^a^IQR

Complications related to mechanical factors such as oxygenator malfunction, circuit clots, and cannula problems were noted in 21.4% (95% CI: 1.6–28.8%) of patients. Bleeding occurred in 15.5% (95% CI: 5.5–43.5%) and infections were seen in 2.6% (95% CI: 0.2–30%) of patients, the majority of which were cannulation site or ventilator-associated infections. Pneumothorax occurring during ECLS was noted in 6.4% (95% CI: 2.8–14.6%) of patients.

## Discussion

This review offers an analysis of the utilization of ECMO and ECCO2R in the management of patients with status asthmaticus who require mechanical ventilation. The findings of this review provide insights into the associated mortality rates and the factors that may influence patient outcomes. Several critical points emerge that warrant discussion (Tables 2 and 3).

**Table 2:** Outcomes

Study	Intervention	*N*	Age	Male	VV	Mortality	MV days	ECLS days
Yeo, 2017 [1]	ECMO	272	36.2 ± 13.4	108 (40%)	250 (91%)	45 (16.5%)	NA	7.4^a^
Zakrajsek, 2023 [5]	ECMO	127	38 ± 13	58 (46%)	105 (83%)	14.6%^b^	NA	NA
Patel, 2020 [6]	ECMO	22	30 ± 14	7 (32%)	NA	0 (%)	12.6 ± 12.6	6.0 ± 1.10
Mikkelsen, 2009 [7]	ECMO	24	31 ± 12	16 (67%)	14 (86%)^c^	4 (25%)	NA	4.7 ± 3
Di Lasco, 2017 [4]	ECMO	16	50 ± 10.6	8 (50%)	13 (82%)	0 (0%)	NA	12.5 ± 4.9
Bromberger, 2020 [3]	ECCO2r	26	32 (27–40)	13 (50%)	26 (100%)	0 (0%)	4 (2–5)	3 (2–6)

*MV* mechanical ventilation

^a^No SD/IQR/Range

^b^Propensity score matched estimates, non-matched data estimates not reported

^c^ECMO mode data available for 16 patients only (no ECMO mode data for 8 of 24 patients)

**Table 3:** Complication rates

Study	Modality	*N*	Bleeding	GI bleed	Site bleed	Limb ischemia	Mechanical	Infection	Pneumothorax	Dialysis	Cannula thrombus	DVT
Yeo, 2017 [1]	ECMO	272	77 (28)		37 (14)	7 (3)	67 (25%)	45 (17)	14 (5)	54 (20)	0 (0)	0 (0)
Zakrajsek, 2023 [5]	ECMO	127	9(7)	3 (4)	0 (0)	0 (0)	0 (0)	1 (1)	4 (5)	30	0 (0)	0 (0)
Patel, 2020 [6]	ECMO	22	NA	NA	NA	NA	NA	NA	NA	NA	NA	NA
Mikkelsen, 2009 [7]	ECMO	24	9 (38)	0 (0)	6 (25)	0 (0)	10 (42)	2 (8)	0 (0)	3 (13)	0 (0)	0 (0)
Di Lasco, 2017 [4]	ECMO	16	0 (0)	0 (0)	0 (0)	0 (0)	0 (0)	0 (0)	0 (0)	0 (0)	0 (0)	0 (0)
Bromberger, 2020 [3]	ECCOR	26	5 (19)	0 (0)	1 (4)	0 (0)	0 (0)	0 (0)	2 (8)	0 (0)	6 (23)	12 (46)

Firstly, the review underscores the considerable variability in mortality rates among status asthmaticus patients treated with ECMO or ECCO2R. The pooled mortality rate of 13.7% is a notable finding, but it is important to recognize that the included studies demonstrated a wide range of mortality rates from 0 to 26%. This substantial variation highlights the need for a more nuanced understanding of the factors contributing to patient outcomes within this population. A significant limitation observed in the studies analyzed is the scarcity of comprehensive data on pre-ECMO lung mechanics. Parameters such as airway pressures, intrinsic positive end-expiratory pressure, tidal volumes, and driving pressures, which are central to the pathophysiology of asthma and its management, were not consistently reported. The absence of these crucial data complicates the interpretation of outcomes of ECMO and limits selection of patients who would benefit from escalation to ECMO vis-à-vis mechanical ventilation.

Despite the lack of detailed data on lung mechanics, a multivariate analysis by Yeo et al. [1] identifies PEEP as the pre-ECMO variable associated with post-ECMO mortality. This emphasizes the potential importance of PEEP levels as a predictive factor in risk assessment and treatment planning for patients with status asthmaticus. With regard to complications while on ECMO, the relative risk of mortality increased threefold with cannulation site bleeding (OR, 2.94, 95% CI, 1.35–6.41, *p* = 0.007), sixfold with pulmonary bleeding (OR, 5.79, 95% CI, 1.92–17.44, *p* = 0.002) and fourfold with central nervous system bleeding (OR, 3.93, 95% CI, 1.19–12.99, *p* = 0.025) [1]. Bleeding occurred in 28% of patients in the ELSO registry (95% CI 23–34%) and varied across other studies from 0 to 37%. ELSO data also showed higher mortality with multiorgan damage, which may result from hemodynamic consequences of severe hyperinflation, ECMO-related bleeding, or concurrent sepsis. Fourteen of 127 patients started on VV ECMO but switched to VA ECMO, while 5 patients required VA ECMO as the initial therapeutic modality. Compared to ECMO, an ECCO2R study by Bromberger et al. [3] reported 15% bleeding and 100% survival. This may be due to the small cannula and blood flow requirements for ECCO2R and, therefore, may be a safer alternative and thus should be further investigated. Surprisingly, severe respiratory acidosis and elevated peak airway pressures, which are often indicative of the severity of asthma, were not found to be associated with post-ECMO mortality in the ELSO database [1]. This discrepancy suggests that additional factors beyond these baseline physiological parameters may be influencing mortality in status asthmaticus patients undergoing ECMO or ECCO2R.

It is essential to acknowledge the limitations of this review, which include significant heterogeneity among the included studies and potential selection biases. The absence of standardized criteria for ECMO initiation and the lack of randomized comparisons with mechanically ventilated patients present challenges in drawing definitive therapeutic decisions.

In conclusion, this review suggests that ECMO and ECCO2R may reduce mortality in mechanically ventilated status asthmaticus patients compared to historically reported mortality rates with mechanical ventilation alone. However, these findings should be interpreted cautiously in light of the limitations inherent in the included studies. To address these limitations and provide more robust evidence, future research should focus on standardized criteria for ECMO initiation, direct comparisons with mechanically ventilated patients, and the development of well-designed prospective studies and registries that can correlate pre-ECMO lung mechanics with post-ECMO outcomes. Such efforts will be instrumental in identifying the status asthmaticus patient population that can benefit most from these potentially life-saving, albeit invasive, modalities.
